# Socio-demographic characteristics and risk factors for HIV transmission in female bar workers in sub-Saharan Africa: a systematic literature review

**DOI:** 10.1186/s12889-020-08838-8

**Published:** 2020-05-15

**Authors:** Peter Dambach, Bathsheba Mahenge, Irene Mashasi, Aisa Muya, Dale A. Barnhart, Till W. Bärnighausen, Donna Spiegelman, Guy Harling

**Affiliations:** 1 Institute of Global Health, University of Heidelberg, Heidelberg, Germany; 2 College of Health Sciences, University of Dodoma, Dodoma, Tanzania; 3 Management and Development for Health (MDH), Dar es Salaam City Council, Dar es Salaam, Tanzania; 4 Department of Epidemiology, Harvard T.H. Chan School of Public Health, Boston, MA USA; 5 Department of Global Health and Population, Harvard T.H. Chan School of Public Health, Boston, MA USA; 6 Africa Health Research Institute, KwaZulu-Natal, South Africa; 7 Department of Biostatistics and Nutrition Harvard T.H. Chan School of Public Health, Boston, MA USA; 8 Center for Methods of Implementation and Prevention Science, Yale School of Public Health, New Haven, CT USA; 9 Department of Biostatistics and Center for Methods on Implementation and Prevention Science, Yale University, New Haven, USA; 10 Institute for Global Health, University College London, London, UK; 11 MRC/Wits Rural Public Health & Health Transitions Research Unit (Agincourt), School of Public Health, University of the Witwatersrand, Johannesburg, South Africa; 12 Harvard Center for Population and Development Studies, Harvard T.H. Chan School of Public Health, Cambridge, MA USA

**Keywords:** Barmaids, Bar girls, Waitresses, Sex work, Behavioral risk factors, HIV, Sub-Saharan Africa

## Abstract

**Background:**

Although sex workers are considered a key population in the HIV epidemic in sub-Saharan Africa (SSA), less consideration has been given to female bar workers (FBW), whose primary occupation is not sex work but who often engage in transactional sex. Understanding FBWs’ risk profiles is central to designing targeted HIV prevention interventions for them. This systematic review describes the socio-demographic characteristics and risk factors for HIV transmission among FBWs in SSA.

**Methods:**

We searched six databases: PubMed, Google Scholar, Web of Science, Popline, Embase and additionally the World Health Organization’s WHOLIS database for grey literature between July and September 2017. Inclusion criteria were reporting (1) primary socio-demographic or behavioral data; on (2) women who sold or delivered drinks to clients; (3) in establishments serving alcohol; (4) in SSA. We excluded studies not presenting separate data on FBWs. We extracted quantitative and qualitative data from the selected studies and conducted a qualitative synthesis of findings.

**Results:**

We found 4565 potentially eligible articles, including duplicates. After applying inclusion and exclusion criteria, we retained 19 articles. FBWs often migrated from rural to urban areas due to economic need or social marginalization. They began bar-based transactional sex due to low wages, peer pressure and to increase financial independence. FBWs had high HIV risk awareness but low agency to negotiate condom use, particularly with regular partners or when offered higher prices for condomless sex. FBWs were also vulnerable to violence and stigmatization.

**Conclusions:**

FBWs are a vulnerable population for HIV infection. Despite social stigmatization and elevated risk of contracting STIs, bar work remains attractive because it enables unskilled women to both, make a living and maintain some independence. FBWs face HIV-related risk factors at the individual, community and societal level and may benefit from biomedical, behavioral and structural interventions.

## Background

Female bar workers (FBWs) in sub-Saharan Africa (SSA) are women who sell or deliver drinks to customers in commercial establishments. Many women work as FBWs: in some countries of SSA, bars may be among the major sources of paid employment for young women [1]. Payment schemes for FBWs vary: while fixed monthly salaries are common in high-end bars, elsewhere FBWs often do not receive fixed wages but instead are paid by the number of customers they serve, or in tips [2, 3]. The low wages, coupled with peer pressure from colleagues and matrons (men or women who manage FBWs´ shifts and deals with their clients), pushes many FBWs toward paid or transactional sex, which can be defined as “noncommercial, non-marital sexual relationships motivated by the implicit assumption that sex will be exchanged for material support or other benefits” [4]. While FBWs’ primary occupation is not sex work, and they typically do not identify themselves as female sex workers (FSW) [5, 6], many FBWs exchange sex for money and often generate a considerable part of their income from sex [7].

While some equate FBWs with FSWs [8], it is likely that there are substantial differences in the groups’ risk factors for HIV acquisition and transmission. Although FSWs and FBWS may share similar social backgrounds and life histories, compared to FSWs, FBWs report relatively short tenure in their profession and lower frequencies of paid sex acts [9, 10]. HIV prevalence in FBWs has previously been found to be correspondingly lower than in FSWs [9]. It is therefore important to consider FBWs risk profiles separately from those of FSWs.

**Fig. 1:**
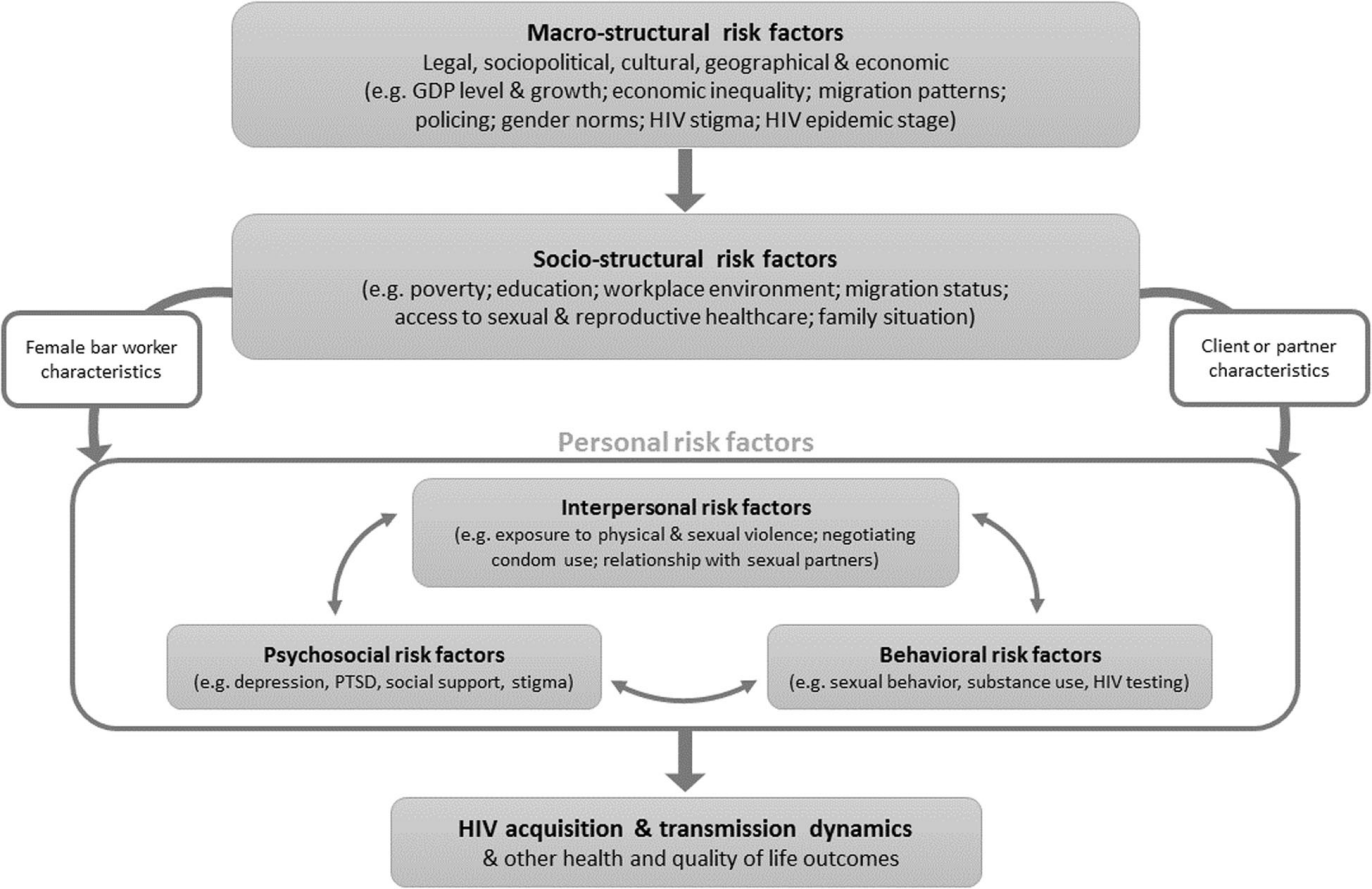
Structural HIV determinants for female bar workers (modified from Shannon et al. [11]

There are a range of risk factors for HIV acquisition and transmission among FBWs. Following from the work of Shannon and colleagues for FSWs [11], we conceptualize these risk factors as falling into three broad categories (Fig. 1). First, macro-structural factors acting at the societal level, including the legal, political, cultural and economic situation of the country or region under study. Second, socio-structural factors that form FBWs´ immediate context, including their poverty level, educational status, healthcare access and workplace environment. Finally, personal factors specific to each FBW, including interpersonal factors such as the nature of their relationship with sexual partners, exposure to gender-based violence, behavioral factors such as sexual behaviors, substance use and healthcare seeking; and psychosocial factors including depression and experience of stigma.

Empirical research on FBWs in SSA is limited, and when FBWs have been surveyed, they are often treated as a subgroup of FSWs. While several systematic literature reviews have been published on FSWs for the SSA region [12–17], we are unaware of any for FBWs. In this systematic review, we summarize the available literature on FBWs in SSA. First, we examine information about the socio-structural risk factors that lead women to engage in bar work. Second, we summarize what is known about FBWs’ interpersonal relationships, psychosocial wellbeing, and behaviors with a focus on how these factors may affect FBWs’ risk of acquiring HIV. Finally, we examine available evidence on HIV prevalence and personal life goals.

## Methods

We conducted a systematic review between July and September 2017, following the guidelines laid out in the PRISMA statement [18]. The literature search was performed both electronically and manually in a multi-stage process. The electronic search included the following databases: PubMed, Google Scholar, Web of Science, Popline, Embase and additionally the World Health Organization’s WHOLIS database for grey literature. The manual search was conducted by inspecting reference lists from articles found in the electronic search. No restrictions on time period or language were applied. The search was performed in English but no articles were excluded from full text assessment if published in another language.

The search terms used were from the following categories: (1) female bar workers; (2) HIV; and (3) Africa, and were broadened with related expressions for each topic. For PubMed, we used MeSH-terms for sub-Saharan Africa and HIV; for Google Scholar we performed separate searches including the name of each of 50 sub Saharan African countries, including Sudan. Full details of the search process are provided as Supplementary Content 1.

Results were combined and duplicates removed. First, two authors (PD and BM) independently selected relevant articles from the search results based on titles and abstracts. Disagreements were resolved through mutual consent. All articles retained after the abstract screen went through an independent full-text review by the same two reviewers. Our inclusion criteria were that a study reported: (1) Socio-demographic or behavioral data; on (2) women who sold or delivered drinks to clients; (3) in establishments serving alcohol; (4) in SSA. There was no requirement that bars paid or officially employed these women, so long as the women self-reported providing services other than sex in bars. There was also no requirement that these women self-reported having sex in return for money or goods. We excluded any study that did not separate out data on FBWs from other groups (e.g. cleaning staff, hotel workers, FSWs).

Data from articles meeting the eligibility criteria were extracted into a matrix. We gathered information on the study including study design, duration, sample size, country, region, year of data collection and year of publication. Following our structural HIV determinants framework, we extracted data on socio-structural factors and, where available, interpersonal, psychosocial, and behavioral factors as well as data on HIV serostatus and quality of life outcomes. We summarized data into tables and into a comparative, narrative description. Since the literature included qualitative, quantitative and mixed methods studies, we used a mixed methods approach. Parameters such HIV prevalence, age and duration of time in bar work were captured quantitatively; other factors, such as FBWs´ reasons for entering bar work, their experiences of stigma and their future aspirations are presented qualitatively. We began by looking at how socio-structural factors led women to start bar work and then ended upon transactional sex. We considered how interpersonal, psychosocial, and behavioral factors interacted to generate risk within these transactional relationships. We finished by evaluating FBWs´ HIV serostatus and their plans for the future.

We assessed the study quality for the quantitative studies and the quantitative part of the mixed-methods study using the guidelines for assessing the risk of bias in cross-sectional survey of attitudes and practices [19]. The qualitative studies did not undergo a quality assessment, since there is no standardized framework available for this, it can be subject to various problems and is not generally recommended [20].

## Results

### Systematic literature review

**Fig. 2:**
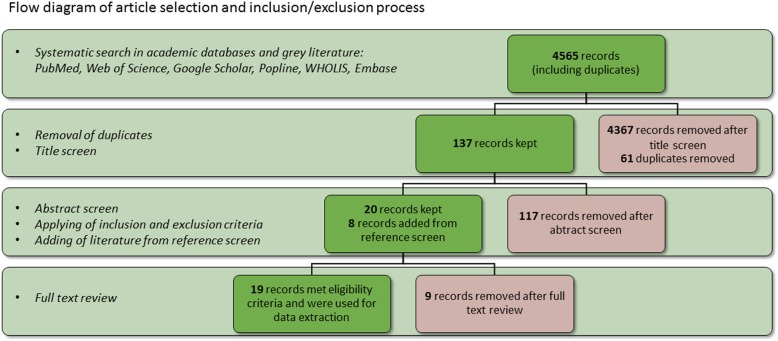
Flow diagram of article selection and inclusion/exclusion process

The initial search identified 4565 articles, including duplicates (Fig. 2). After screening titles and removing duplicates, 137 records were considered for abstract screening. Twenty articles were retained following abstract screen and application of inclusion and exclusion criteria. A review of selected study references found an additional eight potentially eligible publications. All eight additional articles underwent full text review, after which only one article fulfilled inclusion criteria. Two articles, including the newly added one, were excluded from the final review since they were duplicate publications presenting data seen elsewhere [21, 22]. We included 19 references in the systematic review: 15 peer-reviewed research articles, two book chapters, one report and one thesis. Two studies included from Ethiopia [23, 24] and two from Tanzania [7, 10] appeared to present data from the same samples, although this is not specified in the publications. All of the quantitative studies and the quantitative part of the mixed-methods study were of acceptable quality and were hence included in the systematic review. Two further studies were identified through an update of the searches to April 16, 2020 and are presented in the discussion section.

### Study characteristics

The majority of studies were conducted in East Africa: nine in Tanzania, four in Ethiopia, two in Malawi, and two in Uganda; the only West African studies were from Burkina Faso and Ghana Table 1. Seven studies were published before 2000, seven between 2000 and 2009 and five were published after 2010. Only four studies did not specify when their data was collected, but of the remaining 15 studies, only 3 used data gathered after 2005. Eleven studies were entirely qualitative in approach, seven quantitative only and one employed mixed methods. While 7 studies focused exclusively on FBWS, the remaining studies included information on subpopulations of FBWs identified from the general female population (*n* = 2), a mix of FBWs and other workers (*n* = 5), and a mix of FBWs and FSWs (n = 5).

**Table 1:** Characteristics of eligible studies

Author & year	Data period	Type	Country	Location	Study population	Study design	FBW sample size
**Nagot 2002** [9]	1998–2000	PRA	Burkina Faso	Bobo Dioulasso	FSWs incl. FBWs	Cross-sectional survey	67
**Van Blerk 2007** [24]	2003	PRA	Ethiopia	Addis Ababa; Nazareth	FSWs incl. FBWs	FGD; IDI; observation	25
**Van Blerk 2008** [23]	2003	PRA	Ethiopia	Addis Ababa; Nazareth	FSWs incl. FBWs	FGD; IDI; observation	25
**Van Blerk 2011** [25]	2003	PRA	Ethiopia	Nazareth	FBWs who have sex for money	FGD; IDI	30
**Sori 2012** [3]	–	PRA	Ethiopia	Addis Ababa	Women	Cross-sectional survey; FGD; IDI	Quantitative: 284 Qualitative: -
**Messersmith 2014** [6]	2012–13	Report	Ghana	Kumasi	FBW, bar patrons	FGD, IDI	36
**Kishindo 1995a** [26]	1992	PRA	Malawi	Blantyre; Lilongwe; Mzuzu; Zomba	FBW	Cross-sectional survey	540
**Kishindo 1995b** [8]	1992	PRA	Malawi	Zomba	FBW	IDI	30
**Mhalu 1991** [27]	1989	PRA	Tanzania	Dar es Salaam	FBWs; MBWs	Cross-sectional survey	347
**Mnyika 1995** [28]	–	PRA	Tanzania	Arusha; Babati; Moshi; Same	General incl. FBWs	Cross-sectional survey	95
**Talle 1995** [7]	1993	Book chapter	Tanzania	Namanga	FBWs	IDI	30
**Talle 1998** [10]	–	Book chapter	Tanzania	Namanga	FBWs, MBWs	IDI; observation	30
**Mgalla 1997** [29]	1993–95	PRA	Tanzania	Magu district	FBWs	IDI; FGD	33
**Riedner 2003** [30]	2000	PRA	Tanzania	Mbeya region	FBWs	Cross-sectional survey	600
**Akarro 2009** [1]	2004–05	PRA	Tanzania	Dar es Salaam; Mbeya; Zanzibar	FBWs	Cross-sectional survey	2820
**Beckham 2013** [31]	2012	Thesis	Tanzania	Iringa & Njombe regions	FSWs incl. FBWs	IDI; FGD	30 FSW
**Ostermannn 2015** [32]	2012–14	PRA	Tanzania	Moshi	FBWs; male porters	Cross-sectional survey	162
**Gysels 2002** [2]	–	PRA	Uganda	Town in south-west	FSWs incl. FBWs	Life stories	12
**Ntozi 2003** [33]	1999	PRA	Uganda	Kabale; Kampala; Lira	FBWs; FSWs; truck drivers	FGD; IDI	8–12

*PRA* Peer-reviewed article. *FSW* Female sex worker; *FBW* Female bar worker; *MBW* Male bar worker; *IDI* In-depth interview; *FGD* Focus group discussion. Missing data is indicated by “-”

### Socio-structural risk factors

#### Social background

FBWs´ ages vary substantially. Most of the studies reported average ages of around 25 years [1, 9, 29, 30], but FBWs in Malawi [26] and Ethiopia [24] were markedly younger (mean age of 19 years in Malawi, and 14–19 years in Ethiopia, compared to mean age of 25 in Ghana and two studies in Tanzania). Given how long FBWs had already worked in bars, the age at which many of them entered bar work would have been considerably lower, often under 18. Although most FBWs came from economically disadvantaged backgrounds, their school attainment was often above the rural average [26]: several Tanzanian studies reported between 63 and 77% of FBWs had completed primary education [1, 7, 29–31]. Educational attainment was low in Ethiopia and Burkina Faso, reflecting lower overall educational levels in these countries, while keeping in mind the comparably early periods of study conduct in 2000 and 1992 respectively. Although no quantitative proportion among the study population was given in any of the studies, dropping out of school was a precursor of young women migrating to urban areas and initiating bar work that was mentioned in four studies [2, 3, 8, 26]. In Malawi, the most common reasons for dropping out of school were lack of money for school fees, followed by repeated failure to attain the next class in school, and being required to help with domestic labor, often physically exhausting farm work [8, 26]. Two studies cited unintended pregnancy as the trigger of a sequence of events leading to school drop-out [2, 3].

#### Migration

Across all countries, almost no FBWs grew up in the city in which they worked. All ten studies providing information on migration indicated that most FBWs within the respective study populations worked far from their familial home [3, 7–10, 23, 26, 29, 30], in some cases in a different region or even country [29]. Women gave many reasons for migrating. Financial need was one common factor that was explicitly stated in eight studies: many FBWs grew up in poor, rural families with fathers who were subsistence farmers or made a living from basic craftsmanship, and who could not support them [8, 23]. FBWs migrated to find better job opportunities and to avoid physically demanding agricultural work [3, 8–10, 26, 29] (see Table 2). A second factor was the desire for independence: FBWs migrated in order to escape traditional female roles [7] or to escape gender-based violence from abusive partners [23]. In Ethiopia, this violence included female genital mutilation and forced marriage at a young age [23, 25]. Sometimes FBWs migrated because they had been disowned by their parents due to a pre-marital pregnancy or abortion, or because they engaged in socially unacceptable behaviors such as smoking marijuana [23, 25, 26]. Finally, some FBWs moved specifically because they did not want their employment status to be disclosed to their local social network [9, 25, 29].

**Table 2:** Findings on socio-structural risk factors

Author Year Country	Socio-demographics	Factors promoting entry into bar work	Bar environment
Nagot 2002 Burkina Faso [9]	Ages 16–34; 36% foreign; 54% illiterate	–	–
Van Blerk 2007 Ethiopia [24]	Ages 14–19	Poverty	Many FBWs in debt to bar owner
Van Blerk 2008 Ethiopia [23]	Almost all migrated from other regions	Family financial need; escape from FGM & early marriage; disowned by family	Some clients give money without sex
Van Blerk 2011 Ethiopia [25]	Most migrated from rural areas If in hometown, work in bars far from home	–	Current economic situation depends on type of bars they work in
Sori 2012 Ethiopia [3]	Often migrated from rural areas; 32% currently married	Early marriage leading to divorce leading to poverty	
Messersmith 2014 Ghana [6]	Mean age 25; 47% completed high school; 75% unmarried; 19% married/cohabiting 6% divorced	Poverty	Verbal and emotional abuse at bars common
Kishindo 1995a Malawi [26]	Mean age 19; 5–8 years of education; 10% previously married, none now; 12% have children; 97% migrated from rural areas	Economic need for self or family; minority are looking for husband	FBWs are highly mobile and change between bars
Kishindo 1995b Malawi [8]	Mean age 22; all have some formal education & are literate; almost all migrated from rural areas	To earn money (87%), incl. For school fees, to support family; to meet man with good job; unintended pregnancy	–
Mhalu 1991 Tanzania [27]	–	–	–
Mnyika 1995 Tanzania [28]	–	–	–
Talle 1995 Tanzania [7]	Mean age 20; most finished primary school; all single or divorced; many had teen pregnancy	Economic need; independent lifestyle; escape from gender roles Typically poor but not the poorest prior to bar work	–
Talle 1998 Tanzania [10]	Education rate higher than average; most have multi-ethnic background	Economic need; following friend/relative; freedom to make decisions; escape from rural life	1 year post-interview, 90% had changed work place
Mgalla 1997 Tanzania [29]	Mean age 25; 80% have 5 years of education; Half migrated to district; 50% single, 50% divorced; most have children	Economic need; boredom; family troubles; left school due to pregnancy, illness, poverty or forced marriage	Bar business models vary: some pay FBWs wages, others do not
Riedner 2003 Tanzania [30]	Mean age 25; 54% attended secondary school; 21% living with partner, 44% widowed /divorced	–	–
Akarro 2009 Tanzania [1]	Modal age 20–24; 73% attended primary school; 81% single, 17% separated; 70% have children	–	–
Beckham 2013 Tanzania [31]	60% aged 20–29, 40% aged 30–39; 77% primary schooling; 57% single, 33% divorced /separated, 10% widowed; 90% have children	–	–
Ostermannn 2015 Tanzania [32]	–	–	–
Gysels 2002 Uganda [2]	Mean age 30; Marriages: mean of 2, if over 35 mean of 3.5; median of 2 children	Poverty; family troubles; early, often forced, sex leading to pregnancy; easier than farm work	–
Ntozi 2003 Uganda [33]	Age range 15–30	–	–

*BW* Bar work; *CAGE* Cut Down, Annoyed, Guilty and Eye Opener (alcohol use screening test); *FBW* Female bar worker; *FGM* Female genital mutilation; *SW* Sex work. Cells marked “-” were not addressed by the study in question

#### Family

Several studies reported that most FBWs were either single or divorced [1, 7, 8, 26, 29, 31], although two studies, one in Ethiopia the other in Tanzania, found a substantial minority of FBWs were living with husbands or other partners [3, 30] (see Table 2). Nevertheless, many FBWs have children who are partially or fully dependent upon them; several studies reported that the majority of FBWs have one or more children [2, 7, 29]. Those children originated from relationships with former partners, husbands or clients, and, in a few cases, from rape [2]. FBWs often raised these children alone without financial support from the fathers. FBWs’ role as financial providers often extended to other dependents, including parents, siblings and close relatives, for whom they supplement farm incomes and contribute to school fees [10, 23, 26, 29].

#### Entry to bar work

Girls and young women reported starting bar work for a number of reasons. Economic necessity was explicitly mentioned in half of the studies and could be identified from context as a contributor in most others. Bar work was seen as an attractive option for women who lacked opportunities for further education and otherwise would have limited opportunities in the unskilled labor market [29]. Although bars often pay FBWs little or no direct wages for serving drinks, [2, 3, 10, 29], becoming an FBW generally improved women’s economic status through tips and by providing them with opportunities to engage in transactional sex, which allowed women to meet their basic needs and support dependents [7, 10]. Bar work also provided women with the opportunity to meet wealthy partners who they hoped would eventually marry them [31]. In some cases, FBWs left other jobs with more limited economic potential, such as housemaids, cleaners and nannies to work in bars [8, 26]. Friends who were already working as FBWs and becoming financially successful through transactional sex often influenced women to initiate bar work [6, 10].

#### Workplace environment

The bar environment was reported to provide a combination of risks and protective factors, e.g. being subject to violence from bar matrons and other FBWs and sometimes forced alcohol consumption [25]. Sex with bar proprietors is not uncommon [6]. Protection from client violence is often provided by the particular setting of where sex is performed. In many cases, in low-end bars, there are adjacent rooms which FBWs can use for sex [3, 8, 24–26]. In some cases, those rooms were free for the FBWs, in others, the clients were required to pay an additional fee. In contrast, in higher-end bars, wealthy customers may invite FBWs for dinner and take them to a hotel [8, 25]. While clients who take FBWs away from the bar tend to pay more, such movement changes the transaction’s dynamics. Going to a hotel room is often only possible after the FBW’s shift has ended, often late at night. Moving away from the bar can also expose FBWs to risks including physical violence, rape and client non-payment [25].

### Personal risk factors

#### Bar work tenure

Although some FBWs reported having worked in bars for many years, the majority considered bar work as a temporary occupation. In urban Burkina Faso, almost 75% of FBWs were working in their profession for less than 5 years and 30% for less than 1 year [9]. FBWs also reported frequently changing workplaces, often within the same town or city [3, 10, 30]. At Namanga, on the Kenya-Tanzania border, 90% of FBWs had changed their workplace after 1 year [10]. Across studies, the most frequent causes of workplace changes were problems with the bar owner, bar matron or other FBWs.

#### Engagement in transactional sex

There was wide variation in the proportion of FBWs reporting or reported to be engaging in sex work. While in one study [6], none of the FBWs reported engaging in sex work, several other studies state that virtually all FBWs performed transactional sex [7, 9, 26, 29] and most of the FBWs´ relationships involve payment or other forms of benefit [30]. One study reported that transactional sex was also sometimes seen both, as a way to earn money and as a source of pleasure [2]. Only seven studies reported FBWs’ number of sexual partners. These values ranged from 3.3 per week in Burkina Faso [9] to 11 per week in Malawi [26] (see Table 3). All seven studies differentiated between casual and regular clients [2, 7, 8, 25, 26, 29, 33]. Casual clients are often one-time acquaintances, while regular clients have a preferred FBW whom they contact for sex regularly. Casual clients are almost exclusively picked up at the bar, since this is the first point of interaction. If clients are trustworthy and pay well, regular contact might be established. While regular clients are often acquired at the bar, subsequent transactional sex with these clients often occurs elsewhere, usually not at clients’ homes since many of them have families. Regular clients are often married to other women and are more likely to engage in long term relationships with FBWs [29]. They are also more likely to lead to a non-transactional relationship or marriage with the FBW herself [25]. Regular clients are often perceived as more trustworthy and as less of a risk regarding HIV [3, 24]. The benefit of increased income security from a regular client often comes at a cost: First time clients often tend to pay more and in cash and are therefore often preferred over regular ones [33].

**Table 3:** Findings on personal-level risk factors, including behavioral, interpersonal, and psychosocial factors

Author Year Country	Time in bar work	Sex work and type of clients	HIV risk awareness and willingness to test	Condom use	HIV risk magnifiers
Nagot 2002 Burkina Faso [9]	Median of 6 years, with wide range	Mean of 3.3 clients per week	Self-assessment of acquiring HIV in the future. Yes: 31%; No: 31%; Don’t know: 37%	12% of sex acts condomless	–
Van Blerk 2007 Ethiopia [24]	Timespan worked in bars often short	SW venue: back rooms of bars; Mean of 1 client per night most nights	High HIV risk awareness	High condom use with clients almost never with boyfriends	Alcohol consumption among FBWs lowers their control over condom negotiation
Van Blerk 2008 Ethiopia [23]	–	–	High risk awareness but feeling powerless to insist on condom use Many afraid to test for HIV	Forced condomless sex or condom cutting common	Several reports of client violence against FBWs
Van Blerk 2011 Ethiopia [25]	–	SW venue: varies by bar: luxury hotel rooms to backrooms and corridors; Regular clients can become boyfriends		Forced condomless sex, sometimes cutting of condoms	Expected to drink alcohol; Socially isolated if friends & family know occupation
Sori 2012 Ethiopia [3]	Mean of several years; Frequent bar changes	Expectation that low/no wages supplemented by SW; SW venue: back room of bars		Condom use occasional with new clients, rare for regulars	
Messersmith 2014 Ghana [6]	–	Few admitted doing transactional sex; widespread agreement that sex work is done by FBW	High HIV knowledge; 64% FBWs think they are at risk of HIV, most due to partner infidelity 64% had ever tested for HIV	–	Low levels of alcohol use reported; client alcohol use seen as risky; violence largely IPV, not at bars
Kishindo 1995a Malawi [26]	Mean 3 years	Expected to make money from SW not BW; mean of 11 clients per week; SW venue: back rooms of bars	Low HIV risk awareness	23% lifetime condom use	Willing to not use condoms with clients if enough money paid Stigmatized because they are perceived to spread HIV
Kishindo 1995b Malawi [8]	All consider BW a temporary occupation;	SW venue: in back room, or elsewhere post- shift; 40% report regular clients.	High awareness that multiple partners risky; low HIV risk self-perception	30% lifetime condom use	–
Mhalu 1991 Tanzania [27]	–	–	–	Unmarried and longer term FBWs with access to counseling had condom use rates of around 65%	–
Mnyika 1995 Tanzania [28]	–	92% of FBWs are aware of the importance of condom use	–	47% lifetime condom use; 44% always have condoms available; 28% regularly insist on condom use with clients	–
Talle 1995 Tanzania [7]	–	All FBWs engage in SW; often have clients & boyfriends	High awareness, low perceived self-risk for HIV	Condom use rare	Reports of violence if refuse client’s demanded sexual practices Asking clients to use condoms raises stigma
Talle 1998 Tanzania [10]	All working in Namanga < 1 year	Number of clients answered imprecisely & perhaps underreported	–	–	–
Mgalla 1997 Tanzania [29]	–	Almost all have both casual & regular clients	High but imperfect HIV awareness	Some condom use with casual clients, none with regulars;	Low negotiation power
Riedner 2003 Tanzania [30]	–	75% of FBWs´ relationships involve money	–	Condom use ~ 50% ever with both, regular & casual partners	–
Akarro 2009 Tanzania [1]	–	–	High HIV risk awareness	90% report more money for condomless sex	96.7% regularly drink alcohol
Beckham 2013 Tanzania [31]	–	–	High HIV risk awareness Reports of FBWs unveiling their occupation in front of doctors to receive appropriate care	Condom decisions often left to client; condomless sex better paid	Stigmatized if reveal their profession to HCW; often assumed HIV-positive & referred to ART clinic
Ostermannn 2015 Tanzania [32]	–	FBWs have twice as many lifetime sexual partners as non-FBW peers	FBWs willing to test for HIV when tests are required by officials	–	FBWs less willing than others use home-based HIV testing, more concern re. disclosure
Gysels 2002 Uganda [2]	–	Women have both casual & regular clients Some claim to enjoy work: can make money & avoid demands for sex from local men; some do well through SW, others still struggling	High HIV risk awareness	Condom use acceptable with casual, but taboo for regular clients	Alcohol use leads to rape; 82% have been subject to violence, 44% to forced sex. Majority of rape prior to starting bar work
Ntozi 2003 Uganda [33]	–	Both types of casual & regular clients; prefer casual because they pay higher and mostly cash	High HIV risk awareness High stated willingness to test for HIV, but no past testing Barriers to HIV testing: cost & no facility	Increased condom use in recent years	Alcohol claimed as reason for multiple partners & low condom use

#### Condom use

FBWs generally reported knowing about HIV, and that condoms can protect against it. Despite this, condom use was low – reported lifetime use in Malawi and Tanzania was between 23 and 50% [26, 30] reflecting different times of study conduct between 1992 and 2000 (see Table 3). Several reasons for limited condom use were mentioned in the studies reviewed. First, FBWs often did not perceive themselves to be at imminent risk, or at greater risk than others, of acquiring HIV [34, 35]; however, in a study from Ghana, 64% of FBWs perceived themselves as vulnerable to HIV [6]. Second, condom use varied by client type: condoms were often used with casual partners, but virtually never with regular clients, who were perceived as being more trustworthy [2]. Third, FBWs sometimes reported being willing to forgo condoms if the clients paid more money [1, 3, 8]. Fourth, some FBWs feared that condoms could remain in the vagina and cause sterility or itchiness [26] or that condoms are impregnated with HIV in order to infect people [29]. Finally, condomless unprotected sex was sometime imposed by clients, and in several studies FBWs reported that clients intentionally broke or removed condoms during intercourse [23, 24, 33].

#### Alcohol and drug use

Heavy alcohol use was reported by FSWs in several studies [1, 10, 33]. One study reported that 96.7% of FBWs regularly drink alcohol and that FBWs who consumed alcohol were almost seven times less likely to use condoms [1]. Alcohol and drug use was reported to hinder condom negotiation and lead to more sexual partners [2, 6, 33, 36] (see Table 3).

#### Stigmatization and violence

FBWs are at high risk for stigmatization and violence. Some reported being treated “as though we are garbage”, and feeling shunned by friends and family once they discover they work in bars [25]. In some cases, disclosure of an HIV-positive status to colleagues led to further stigmatization, bullying and sometimes dismissal from the bar [23]. In one study, FBWs reported often not testing for HIV or delaying seeking treatment because they feared being labeled as prostitutes by colleagues, husbands and the wider society [37]. Some FBWs reported having experienced sexual and physical violence from their clients [2, 23, 25]. One study reported that 82% of FBWs have been subject to violence and 44% to forced sex [2]. Sometimes not agreeing to sexual practices demanded by the client led to violence [7]. Pressure or violence can be exerted by bar owners and other FBWs. None of the studies presented data on the psychosocial outcomes of stigmatization of violence.

#### HIV testing

Six studies provided data on FBWs past use of and willingness to conduct HIV testing. HIV testing was reported to be generally acceptable, although some FBWs reported never having tested because they were afraid of being positive [23]. In a study from Ghana only 64% of FBWs had ever tested for HIV, despite many thinking themselves at increased risk due to frequent partner infidelity [6]. Despite being afraid of testing, Tanzanian FBWs often reveal their occupation (with its concomitant risk of HIV) to doctors in order to obtain appropriate care [31]. The rate of HIV testing was significantly higher among FBWs who were required to participate in a municipality-mandated health screening program [32].

### HIV prevalence

Only three studies reported HIV prevalence rates for their samples (see Table 4): 40% in Burkina Faso [9], and 52 and 68% in two studies from Tanzania [27, 30].

**Table 4:** HIV prevalence and future aspirations of FBWs

Author Year Country	HIV prevalence	Future goals and aspirations
Nagot 2002 Burkina Faso [9]	40% HIV-positive	–
Van Blerk 2007 Ethiopia [24]	–	–
Van Blerk 2008 Ethiopia [23]	–	Bar work discussed as possible aid to transition to adulthood
Van Blerk 2011 Ethiopia [25]	–	Many want own small business & a family with children
Sori 2012 Ethiopia [3]	–	–
Messersmith 2014 Ghana [6]	–	–
Kishindo 1995a Malawi [26]	–	All want to leave work & get married, none sees SW as a lifetime trade
Kishindo 1995b Malawi [8]	–	All FBWs want to get married
Mhalu 1991 Tanzania [27]	52% HIV-positive	–
Mnyika 1995 Tanzania [28]	–	–
Talle 1995 Tanzania [7]	–	–
Talle 1998 Tanzania [10]	–	–
Mgalla 1997 Tanzania [29]	–	Most want to change jobs, many want to be small-scale traders
Riedner 2003 Tanzania [30]	68% HIV-positive	–
Akarro 2009 Tanzania [1]	–	–
Beckham 2013 Tanzania [31]	–	
Ostermannn 2015 Tanzania [32]	–	–
Gysels 2002 Uganda [2]	–	–
Ntozi 2003 Uganda [33]	–	–

### Life goals

Despite this high HIV prevalence, bar work was often perceived as a first step towards a better life [7]. Almost all FBWs aspired to have their own family with children [8, 26]. Many stated they were trying to put money aside to start their own small business as petty traders [25, 26]. A small proportion of FBWs were saving money to open their own bar, following the example of former FBWs for whom transactional sex coupled with strong entrepreneurial skills became a stepping stone for starting their own bars and becoming financially independent [2].

## Discussion

Women working in bars in SSA are a socio-economically underprivileged group, many of whom engage in transactional sex. FBWs make up a larger part of the population than do FSWs [1], but to date have received little attention, as reflected in the small number of studies in our systematic review. Within our review, we found that FBWs share several characteristics with FSWs, including high social stigma, being subject to violence, and having elevated sexual behavior risks. Differences between FBWs and FSWs often arise less from the presence or non-presence of specific factors, but rather from their severity. This is reflected, ultimately, in FBWs´ elevated HIV prevalence compared to the general population, but lower than FSWs [9].

Literature on FBWs´ HIV acquisition risk is scarce for other parts of the world, potentially because FBWs are not recognized as conducting sex work in the same way as in SSA. However, past reviews have suggested that sex work contributes a greater attributable fraction of HIV infections in the general female population in SSA (17.8%) than elsewhere [27]; this in turn reflects a higher HIV prevalence among FSWs in SSA (36.9%) than elsewhere [17]. As a result, it seems likely that the FBW HIV prevalence figures found in our review (40–68%) are also substantially higher than would be seen in FBW in other countries. Opportunities to compare the HIV prevalence between studies and other populations, such as the general population or FSWs are limited due to the different decades in which studies were conducted, which is equally the case for behavioral changes such as condom use, that are likely to have changed over the last decades due to increased access, education and awareness campaigns and do not necessarily reflect a difference between study sites at one point in time. Additionally, selection bias during data generation might affect studies on FSWs and FBWs differently to those in the general population. If people already know or assume themselves to be HIV seropositive, then they are less likely to agree to an HIV test, and HIV prevalence will be underestimated [38].

Our review shows that bar work represents a relatively brief period in the life course of many poor rural women. These young women often move to urban areas and start working in bars due to financial need. This financial need is often created or compounded by forced early marriage, abuse or marital breakdown, or having left rural homes to escape boredom or agricultural work. Pre-existing financial need, coupled with limited earnings opportunities as FBWs, often leads to engagement in transactional sex. Although many FBWs already have children, almost all aim to build a family in the future. Most FBWs hope to move into other professions relatively soon; however some of these occupations, such as petty trading, might not necessarily end transactional sex, but rather change its conditions and modalities such as where to pick up clients and where to perform the transactional sex [29].

Reliable information on how many FBWs regularly engage in transactional sex, and how frequently they engage in transactional sex, is difficult to obtain. Our review suggests that some FBWs report having multiple clients each week while others report few or none. None of the studies suggested client numbers as high as those reported by FSWs [17], however some FBWs have multiple sexual partners over short periods of time, putting them at risk of acquiring and transmitting STIs, including HIV. It was also difficult to distinguish transactional sex from other relationships, since there is no clear-cut separation between transactional and non-transactional partners. Indeed, some regular transactional clients may become regular partners and then perhaps even husbands.

Among FBWs engaging in transactional sex, male condom use was insufficient, despite high awareness of its importance in preventing HIV. This discrepancy appears to arise from multiple sources. First, most FBWs do not report perceiving their risk of contracting STIs to be higher than that of the general population. This may reflect a true misperception, or a rationalization of the circumstances that push FBWs towards to not use condoms. Second, FBWs have a limited ability to negotiate safer sex, something that is often compounded by conducting transactional sex in insecure environments such as corridors and backrooms. Safer sex negotiations are further undermined by the reality that condomless sex is substantially better paid. Third, the perception that regular clients and boyfriends are safer, and not asking them to use condoms to not speak out mistrust, mean that sex with these clients is almost always condomless. Finally, the omnipresence of alcohol within the workplace environment leads to lower ability to negotiate condom use and higher risk of violence [1, 2, 33]. No other forms of barrier or non-barrier STI contraception were discussed in the literature reviewed.

To our knowledge there are no published evaluations of HIV prevention interventions focused specifically on FBWs. However, the common themes arising from the literature suggest that similar interventions to protect FBWs against exploitation and acquisition of HIV and other STIs may be useful across countries in SSA [39]. Such interventions can be considered at the various levels of our conceptual framework (Fig. 1). At the structural level, macro-structural interventions, including regulative and protective laws, policies, or socio-structural interventions, such as targeted support programs for FBWs, could provide support for adolescent girls and young women to stay in school and to protect them from gender-based violence in rural areas. Programs could also provide urban women with access to alternate sources of income or occupational training. Enacting and enforcing statutory minimum wages for FBWs might additionally lower the proportion of FBWs engaging in transactional sex once working in bars. Given that a high proportion of FBWs are single mothers, interventions including childcare support, including support for education costs, might also help. There may also be benefit in supporting FBWs to build peer-support organizations. Although peer-support organization has been demonstrated to be successful among FSWs in Africa [38], these interventions are markedly absent from the discussion in FBW literature.

At the personal level, FBWs’ engagement with transactional sex with limited barrier protection appears to be primarily a function of external pressures, such as low income, social stigma, and threat of violence rather than a lack of knowledge of HIV risks and protective options. This suggests that interpersonal interventions might be more important than those focused specifically at FBWs. For example, interventions might focus on working with bar owners/matrons and FBWs’ clients, to increase willingness to provide and use condoms, respectively. Skills training might help FBWs negotiate condom use more effectively, if implemented alongside efforts to change societal and client attitudes. Female-controlled protective measures, such as pre-exposure prophylaxis (PrEP) – which has been shown to be acceptable among FSW and FBW [39–42] – might also be helpful, although adherence may be limited by elevated alcohol consumption and stigma that can be created if FBWs are observed taking pills. It is unclear to what extent psychosocial support is needed, given the dearth of evidence on psychopathology in the literature. Two further studies from Tanzania and Cameroon that were published after completion of the systematic literature search are widely in line with the findings from included studies and do not change the presented risk profile and socio-demographic characteristics of FBW [43, 44]. However, the study from Tanzania [43] provides data showing that HIV prevalence among FBW is not significantly higher than in the general population of Dar es Salaam, and that, in a similar study population of FBW, it is much lower today (7.1%) compared to 30 years ago (52%) [27].

## Strengths and limitations

This systematic review has the strength of including not only peer-reviewed literature, but also one research report, one thesis and two book chapters on FBWs in sub-Saharan Africa, limiting the potential for selection bias. The use of a robust conceptual framework also ensures that we systematically considered risk factors reported in the studies we found. Nevertheless, some factors limit the generalizability of the results we present. Since the majority of studies included were performed in East Africa means that generalizing beyond eastern Africa is difficult. The focus of this study on FBWs means we did not consider other types of informal sex workers, such as women working in markets or fishing villages [45]. However, the majority of included studies does not provide a formal definition of FBWs, which makes epidemiological comparisons difficult. Furthermore, the publications reviewed themselves do not cover some important topics and the lack of data on statistically significant associations between specific risk factors and HIV transmission among FBWs make it difficult to quantify results. The literature’s focus on mid- to low-end bars means we can say little about traditional bars where local brew is served, or high-end hotel settings. Additionally, while violence and stigma are discussed, the psychosocial health of FBWs is virtually unreported. Some of the published literature on this topic dates back several decades and hence does not reflect the current state of HIV prevalence and condom use. Finally, the sparsity of data across space and time means that identifying temporal trends in HIV prevalence or key risk behaviors was not feasible, and additional research may be required to understand the risk profiles of contemporary FBWs.

## Conclusions

Female bar workers experience a distinct set of structural and personal risk factors based on their work environment and client contact, which place them at elevated risk for HIV and STIs. Personal history and economic necessity lead them into bar work and then, in many cases, to transactional sex. FBWs would likely benefit from broader economic, reproductive health, and partner-focused interventions, but data on their personal lives, and psychosocial health remains limited. Further research could examine how to adapt individual health and broader policy interventions to FBWs’ particular needs.

## Supplementary information

**Additional file 1.** Supplementary Content 1: Search terms and hits by database.

**Additional file 2.** Supplementary content 2: Assessment of risk of bias for the quantitative studies/ study part. The assessment follows the methods commentary: Risk of Bias in cross-sectional surveys of attitudes and practices (Agarwal et al. 2017).

## Data Availability

All data generated or analyzed during this study are included in this published article.

## Abbreviations

ART: Antiretroviral Therapy
FBW: Female Bar Workers
FSW: Female Sex Workers
HCW: Health Care Workers
HIV: Human Immunodeficiency Virus
IPV: Intimate Partner Violence
WHOLIS: World Health Organization’s database for grey literature
SSA: Sub-Saharan Africa
STI: Sexually Transmitted Infections

## References

[1] Akarro RRJ (2009). Some factors associated with condom use among bar maids in Tanzania. J Biosoc Sci.

[2] Gysels M, Pool R, Nnalusiba B (2002). Women who sell sex in a Ugandan trading town: life histories, survival strategies and risk. Soc Sci Med.

[3] Sori AT (2012). Poverty, sexual experience and HIV vulnerability risks: evidence from Addis Ababa. Ethiopia J Biosoc Sci.

[4] Stoebenau K, Heise L, Wamoyi J, Bobrova N (2016). Revisiting the understanding of “transactional sex” in sub-Saharan Africa: A review and synthesis of the literature. Soc Sci Med 1982.

[5] WHO (2011). Preventing HIV among sex workers in sub-Saharan Africa: A literature review.

[6] Messersmith L, Beard J, Agyarko-Poku T, Longobardi D, Asafo M, Corneliess C, et al. “I can decide to use the property I have to make money”: HIV Vulnerability of Bar Workers and Bar Patrons in Kumasi, Ghana. Cent Glob Health Dev. 2014; Research Report. https://open.bu.edu/handle/2144/28504.

[7] Talle A (1995). Bar workers at the border. Young people at risk: fighting AIDS in northern Tanzania.

[8] Kishindo P (1995). High risk behaviour in the face of the AIDS epidemic: the case of Bar girls in the municipality of Zomba, Malawi. East Afr Soc Sci Res Rev.

[9] Nagot N, Ouangré A, Ouedraogo A, Cartoux M, Huygens P, Defer MC (2002). Spectrum of commercial sex activity in Burkina Faso: classification model and risk of exposure to HIV. J Acquir Immune Defic Syndr 1999.

[10] Talle A (1998). Sex for leisure: modernity among female bar workers in Tanzania. Anthropol Perspect Local Dev Knowl Sentim Confl Lond Routledge.

[11] Shannon K, Strathdee SA, Goldenberg SM, Duff P, Mwangi P, Rusakova M (2015). Global epidemiology of HIV among female sex workers: influence of structural determinants. Lancet.

[12] Scorgie F, Chersich MF, Ntaganira I, Gerbase A, Lule F, Lo Y-R (2012). Socio-demographic characteristics and behavioral risk factors of female sex workers in sub-saharan Africa: a systematic review. AIDS Behav.

[13] Shahmanesh M, Patel V, Mabey D, Cowan F (2008). Effectiveness of interventions for the prevention of HIV and other sexually transmitted infections in female sex workers in resource poor setting: a systematic review. Tropical Med Int Health.

[14] Cwikel JG, Lazer T, Press F, Lazer S (2008). Sexually transmissible infections among female sex workers: an international review with an emphasis on hard-to-access populations. Sex Health.

[15] Chen L, Jha P, Stirling B, Sgaier SK, Daid T, Kaul R (2007). Sexual risk factors for HIV infection in early and advanced HIV epidemics in sub-Saharan Africa: systematic overview of 68 epidemiological studies. PLoS One.

[16] Lancaster KE, Cernigliaro D, Zulliger R, Fleming PF (2016). HIV care and treatment experiences among female sex workers living with HIV in sub-Saharan Africa: a systematic review. Afr J AIDS Res AJAR.

[17] Baral S, Beyrer C, Muessig K, Poteat T, Wirtz AL, Decker MR (2012). Burden of HIV among female sex workers in low-income and middle-income countries: a systematic review and meta-analysis. Lancet Infect Dis.

[18] Liberati A, Altman DG, Tetzlaff J, Mulrow C, Gøtzsche PC, Ioannidis JPA (2009). The PRISMA statement for reporting systematic reviews and meta-analyses of studies that evaluate health care interventions: explanation and elaboration. J Clin Epidemiol.

[19] Agarwal A, Guyatt G, Busse J. Risk of Bias commentary (cross-sectional surveys of attitudes and practices). Systematic Review and Literature Review Software by Evidence Partners 2017. https://www.evidencepartners.com/resources/methodological-resources/risk-of-bias-cross-sectional-surveys-of-attitudes-and-practices/. Accessed 20 Mar 2019.

[20] Evans D, Pearson A (2001). Systematic reviews of qualitative research. Clin Eff Nurs.

[21] Van Blerk L (2016). Livelihoods as relational Im/mobilities: exploring the everyday practices of young female sex Workers in Ethiopia. Ann Am Assoc Geogr.

[22] Akarro RRJ. Some factors associated with high risk behavior among Bar maids in Tanzania. Curr Res J Soc Sci. 2011;3.

[23] Van Blerk L (2008). Poverty, migration and sex work: youth transitions in Ethiopia. Area..

[24] Van Blerk L (2007). AIDS, mobility and commercial sex in Ethiopia: implications for policy. AIDS Care.

[25] Van Blerk L (2011). Negotiating boundaries: the sex work identities of ‘bar girls’ in Nazareth. Ethiopia Gend Place Cult.

[26] Kishindo P. Sexual behaviour in the face of risk: the case of bar girls in Malawi’s major cities. Health Transition Review. 1995;5.

[27] Mhalu F, Hirji K, Ijumba P, Shao J, Mbena E, Mwakagile D (1991). A cross-sectional study of a program for HIV infection control among public house workers. J Acquir Immune Defic Syndr.

[28] Mnyika KS, Klepp K-I, Kvale G, Ole-King’ori N (1995). Risk factors for HIV-1 infection among women in the Arusha region of Tanzania. JAIDS J Acquir Immune Defic Syndr.

[29] Mgalla Z, Pool R. Sexual relationships, condom use and risk perception among female bar workers in north-West Tanzania. Aids Care-Psychol Socio-Med Asp Aids-Hiv. 1997;9. 10.1080/713613167 9337885

[30] Riedner G, Rusizoka M, Hoffmann O, Nichombe F, Lyamuya E. Baseline survey of sexually transmitted infections in a cohort of female bar workers in Mbeya region. Sex Transm Dis. 2003;79. 10.1136/sti.79.5.382 PMC1744739 14573833

[31] Beckham S (2013). “Like Any Other Woman”? Pregnancy, Motherhood, and HIV among Sex Workers in Southern Tanzania. Dissertation. Johns Hopkins University.

[32] Ostermann J, Njau B, Mtuy T, Brown DS, Mühlbacher A, Thielman N (2015). One size does not fit all: HIV testing preferences differ among high-risk groups in northern Tanzania. AIDS Care.

[33] Ntozi JPM, Ahimbisibwe F, Mulindwa IN (2001). Has the HIV / AIDS epidemic changed sexual behaviour of high risk groups in Uganda?. Afr Health Sci.

[34] Ao T, Sam N, Manongi R, Seage G, Kapiga S (2003). Social and behavioural determinants of consistent condom use among hotel and bar workers in northern Tanzania. Int J STD AIDS.

[35] Ao T, Sam NE, Masenga EJ, Seage G, Kapiga SH. Human immunodeficiency virus type 1 among bar and hotel workers in northern Tanzania: the role of alcohol, sexual behavior, and herpes simplex virus type 2. Sex Transm Dis. 2006. 10.1097/01.olq.0000187204.57006.b3 16505740

[36] Pitso JM (2004). Does alcohol use take away condom use? Qualitative evidence from Selibe Phikwe and Mahalapye town districts, Botswana. Afr J Drug Alcohol Stud.

[37] Rosenheck R, Ngilangwa D, Manongi R, Kapiga S (2010). Treatment-seeking behavior for sexually transmitted infections in a high-risk population. Aids Care-Psychol Socio-Med Asp AidsHiv.

[38] Hogan DR, Salomon JA, Canning D, Hammitt JK, Zaslavsky AM, Bärnighausen T (2012). National HIV prevalence estimates for sub-Saharan Africa: controlling selection bias with Heckman-type selection models. Sex Transm Infect.

[39] Chersich MF, Luchters S, Ntaganira I, Gerbase A, Lo Y-R, Scorgie F, et al. Priority interventions to reduce HIV transmission in sex work settings in sub-Saharan Africa and delivery of these services. J Int AIDS Soc. 2013;16. 10.7448/IAS.16.1.17980. 10.7448/IAS.16.1.17980 PMC3589546 23462140

[40] Luchters S, Chersich MF, Rinyiru A, Barasa M-S, King’ola N, Mandaliya K (2008). Impact of five years of peer-mediated interventions on sexual behavior and sexually transmitted infections among female sex workers in Mombasa, Kenya. BMC Public Health.

[41] Koechlin FM, Fonner VA, Dalglish SL, O’Reilly KR, Baggaley R, Grant RM (2017). Values and preferences on the use of Oral pre-exposure prophylaxis (PrEP) for HIV prevention among multiple populations: a systematic review of the literature. AIDS Behav.

[42] Harling G, Muya A, Ortblad KF, Mashasi I, Dambach P, Ulenga N (2019). HIV risk and pre-exposure prophylaxis interest among female bar workers in Dar Es Salaam: cross-sectional survey. BMJ Open.

[43] Barnhart DA, Harling G, Muya A, Ortblad KF, Mashasi I, Dambach P (2019). Structural, interpersonal, psychosocial, and behavioral risk factors for HIV acquisition among female bar workers in Dar Es Salaam. Tanzania AIDS Care.

[44] Akoku DA, Tihnje MA, Vukugah TA, Tarkang EE, Mbu RE (2018). Socio-economic vulnerabilities and HIV: drivers of transactional sex among female bar workers in Yaoundé. Cameroon PloS One.

[45] Béné C, Merten S (2008). Women and fish-for-sex: transactional sex, HIV/AIDS and gender in African fisheries. World Dev.

