# Supplementary material for: Socio-demographic characteristics and risk factors for HIV transmission in female bar workers in sub-Saharan Africa: a systematic literature review
Source: BMC Public Health. 2020 May 15;20:697. doi: 10.1186/s12889-020-08838-8 (PMC7227324; doi:10.1186/s12889-020-08838-8)
Supplement: Supplementary file 1 — Additional file 1. Supplementary Content 1: Search terms and hits by database. [file 12889_2020_8838_MOESM1_ESM.docx]

# supplementary Materials

Supplementary Content 1: Search terms and hits by database

| Database | Search terms | # of articles |
| --- | --- | --- |
| PubMed ^†^ |  |  |
| #1 | ("HIV"[MeSH] OR "Acquired Immunodeficiency Syndrome"[ MeSH]) AND  "Africa South of the Sahara"[ MeSH] AND  (barmaids OR bar maids OR female bar workers OR female pub workers OR filles de bar OR garconetes OR waitstaff) | 23 |
| #2 | (HIV OR AIDS) AND  Africa South of the Sahara[All] AND  (barmaids OR female bar workers OR female pub workers OR waitresses) | 57 |
| #3 | ("HIV" OR "aids") AND  "africa" AND  ("barmaid" OR "barmaids" OR "female bar workers" OR "waitress" OR "waitresses") | 100 |
| Total | #1 OR #2 OR #3 | 180 |
| Google Scholar |  |  |
| #1 | (HIV OR AIDS OR Acquired Immunodeficiency Syndrome) AND  (Names of all sub Saharan African countries separated by OR) AND  (barmaids OR female bar workers OR female pub workers OR waitstaff) | 3980 |
| Web of Science |  |  |
| #1 | ((barmaids OR female bar workers) AND  Africa AND  (HIV OR AIDS)) | 57 |
| Embase |  |  |
| #1 | (HIV OR AIDS) AND  (female bar workers OR barmaids OR waitresses) | 4 |
| Popline |  |  |
| #1 | (HIV OR AIDS) AND  (female bar workers OR barmaids OR waitresses) | 60 |
| WHOLIS |  |  |
| #1 | (HIV OR AIDS) AND  (barmaids OR female bar workers) | 284 |
| Grand total |  | 4565 |

^†^ All PubMed searches included automatic term mapping. All searches were conducted on all PubMed fields.
