# Supplementary material for: Socio-demographic characteristics and risk factors for HIV transmission in female bar workers in sub-Saharan Africa: a systematic literature review
Source: BMC Public Health. 2020 May 15;20:697. doi: 10.1186/s12889-020-08838-8 (PMC7227324; doi:10.1186/s12889-020-08838-8)
Supplement: Supplementary file 2 — Additional file 2. Supplementary content 2: Assessment of risk of bias for the quantitative studies/ study part. The assessment follows the methods commentary: Risk of Bias in cross-sectional surveys of attitudes and practices (Agarwal et al. 2017). [file 12889_2020_8838_MOESM2_ESM.docx]

**Supplementary content 2: Assessment of risk of bias for the quantitative studies/ study part. The assessment follows the methods commentary: Risk of Bias in cross-sectional surveys of attitudes and practices (Agarwal et al. 2017).**


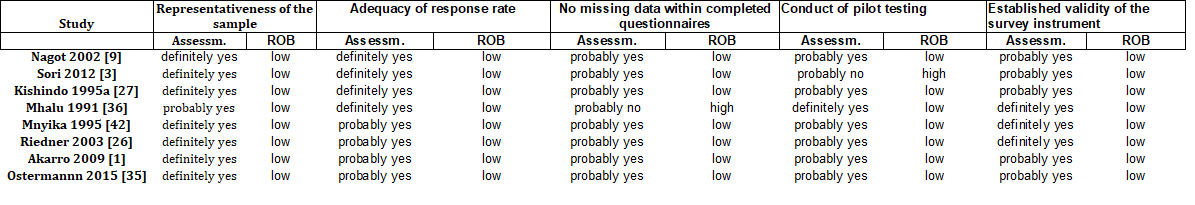
^†^ ROB = Risk of Bias

The assessment for each of the five items is framed as question, and allows four response options for each one: definitely yes (low risk of bias), probably yes (low risk of bias), probably no (high risk of bias), and definitely no (high risk of bias). Response options are framed to facilitate dichotomization of studies as being either “low risk of bias” or “high risk of bias” on an item-by-item basis.
